# Supplementary figures and images for: Backbone Brackets and Arginine Tweezers delineate Class I and Class II aminoacyl tRNA synthetases
Source: PLoS Comput Biol. 2018 Apr 16;14(4):e1006101. doi: 10.1371/journal.pcbi.1006101 (PMC5919687; doi:10.1371/journal.pcbi.1006101)

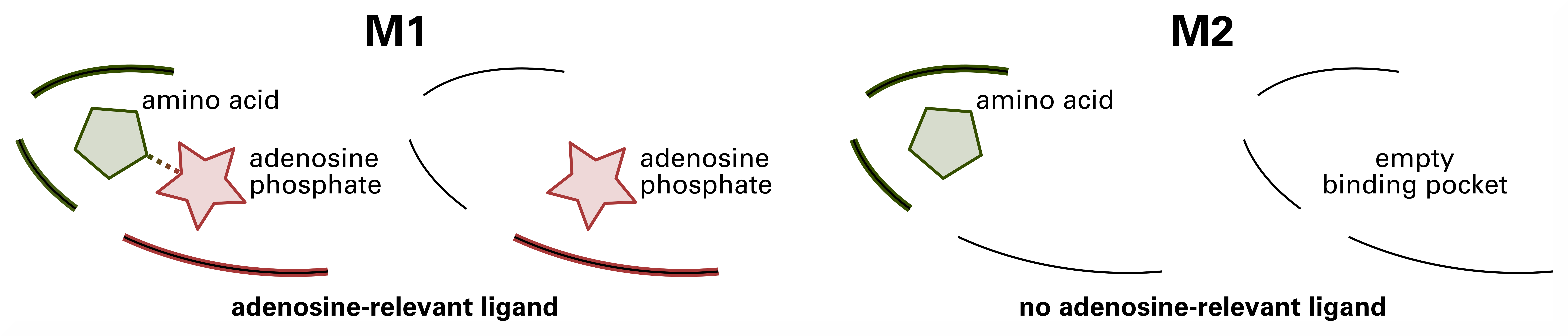

Supplement: S1 Fig — Binding modes M1 and M2 are defined based on the complexed ligand: ligands that bind to the adenosine phosphate moiety (highlighted in red, only in contact when adenosine phosphate is part of the ligand) of the binding site (M1), no ligands or ligands that bind exclusively to the aminoacyl part (green) of the binding site (M2). (TIF) [file pcbi.1006101.s001.tif]

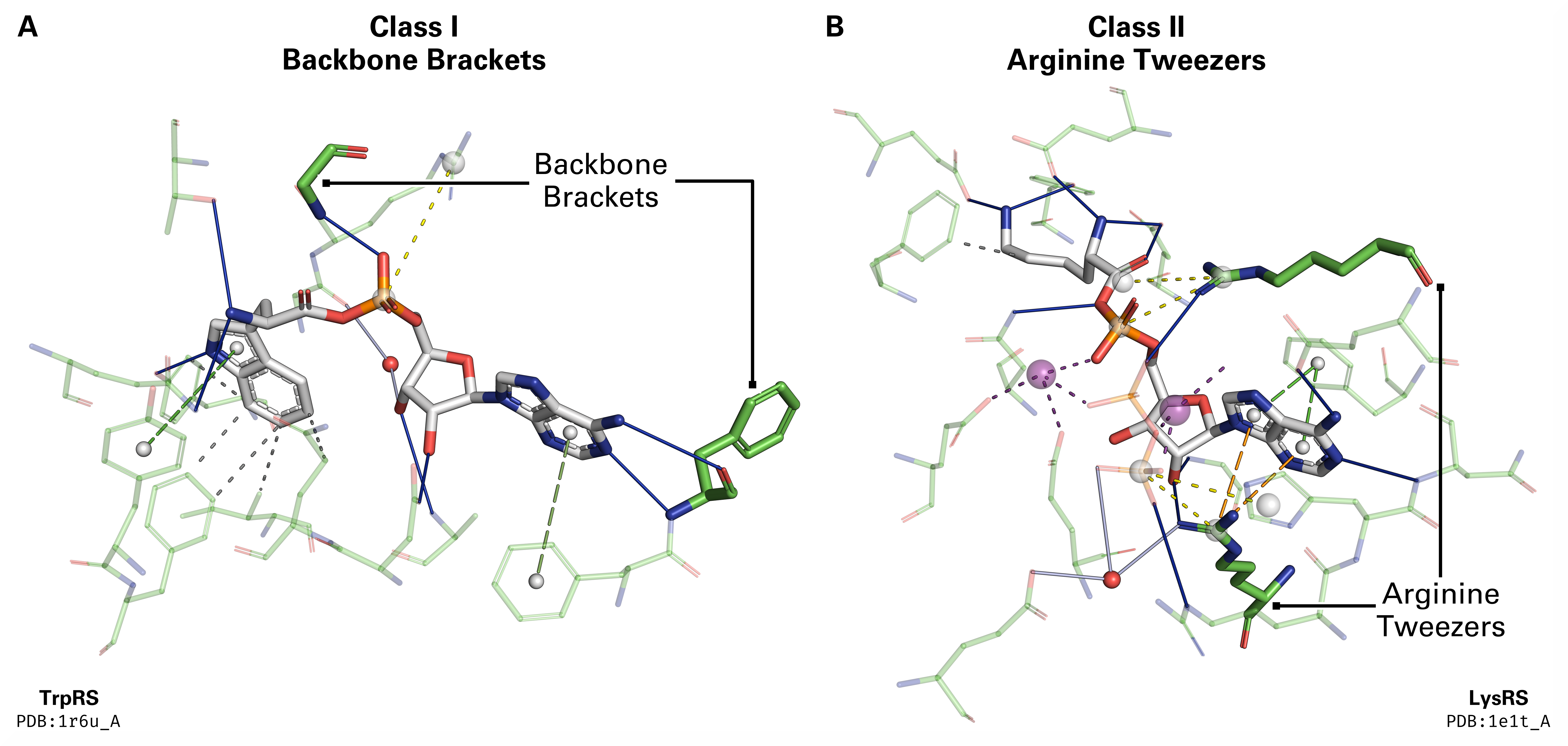

Supplement: S2 Fig — Both aaRS classes contain highly conserved patterns, responsible for proper binding of the adenosine phosphate part of the ligand. Class I aaRS share a highly conserved set of backbone hydrogen interactions with the ligand: the Backbone Brackets. Class II active sites contain a pattern of two arginine residues grasping the adenosine phosphate ligand: the Arginine Tweezers. Interactions were calculated with PLIP [76] and are represented with colored (dashed) lines: hydrogen bonds (solid, blue), π-stacking interactions (dashed, green), π-cation interactions (dashed, orange), salt bridges (dashed, yellow), metal complexes (dashed, purple), and hydrophobic contacts (dashed grey). (A) Class I Backbone Brackets motif and interactions with the ligand Tryptophanyl-5’AMP as observed in TrpRS structure PDB:1r6u chain A. (B) Class II Arginine Tweezers motif and interactions with the ligand Lysyl-5’AMP as observed in LysRS structure PDB:1e1t chain A. (TIF) [file pcbi.1006101.s002.tif]

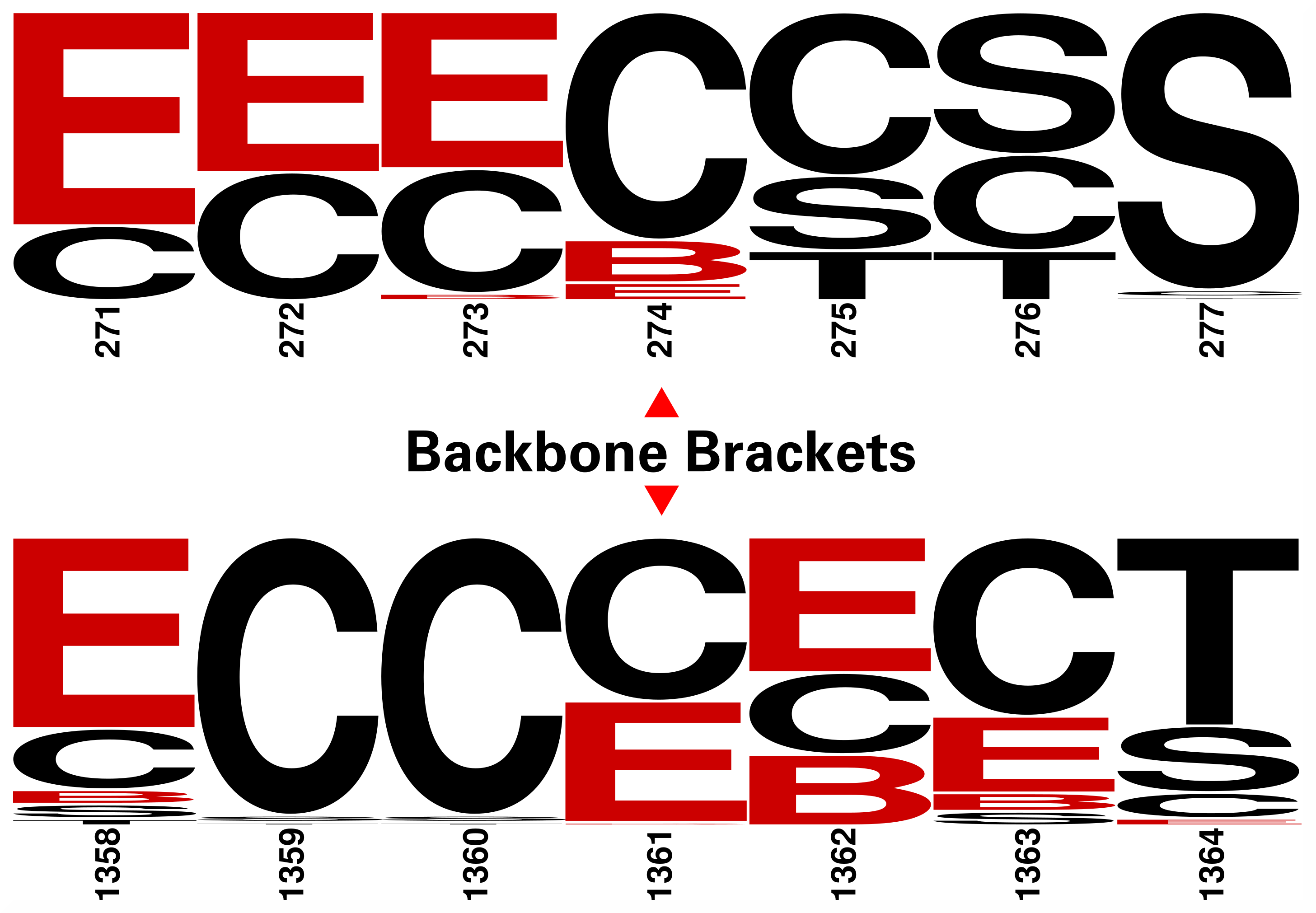

Supplement: S3 Fig — WebLogo [75] representation of secondary structure elements around the Backbone Brackets residues (274 and 1361) annotated by DSSP [123]: helices (blues), strands (red), and unordered (black). Unassigned states are represented by the character “C”. The height of each character corresponds to the relative frequency. (TIF) [file pcbi.1006101.s003.tif]

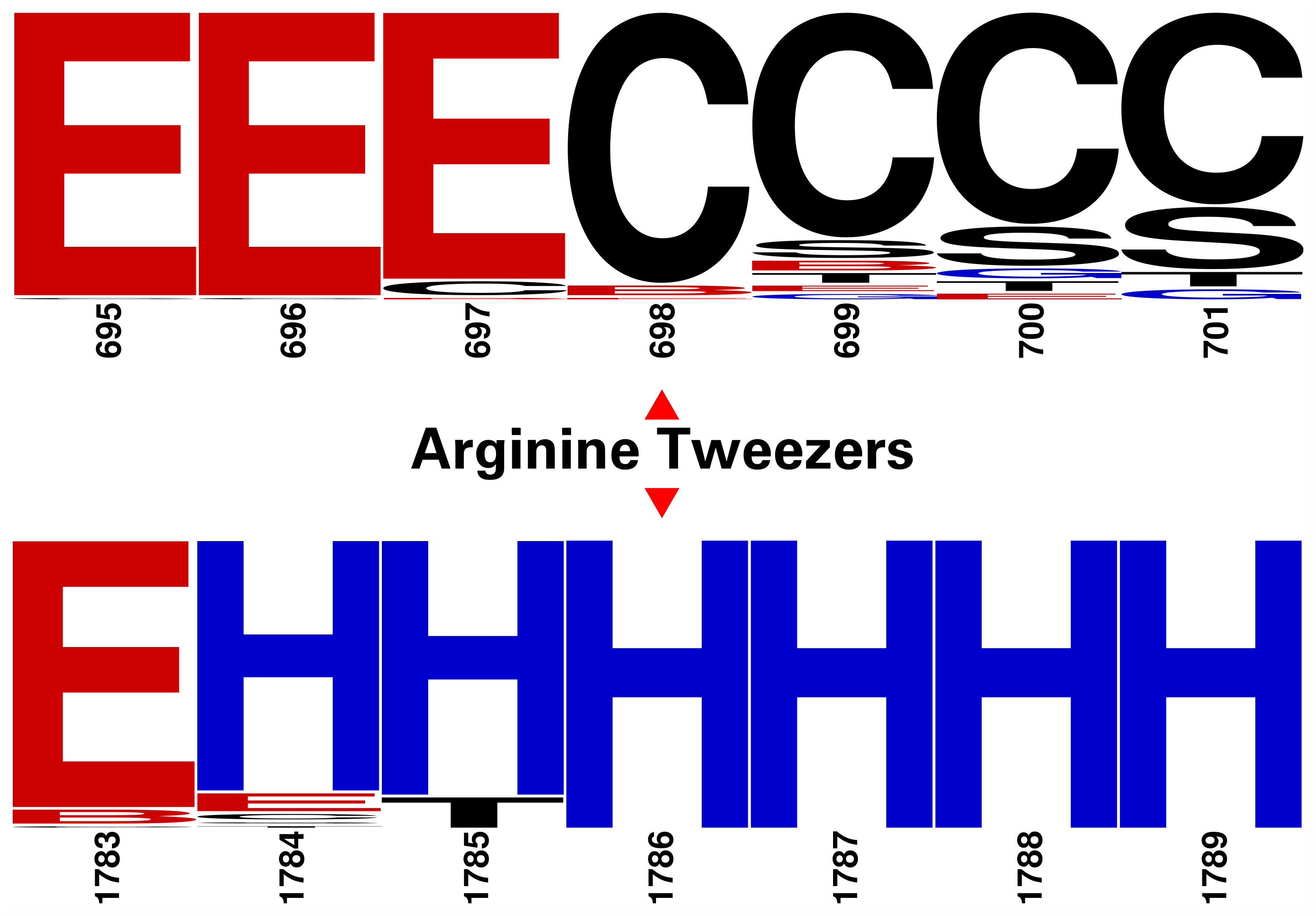

Supplement: S4 Fig — WebLogo [75] representation of secondary structure elements around the Arginine Tweezers residues (698 and 1786) annotated by DSSP [123]: helices (blues), strands (red), and unordered (black). Unassigned states are represented by the letter “C”. The height of each character corresponds to the relative frequency. (TIF) [file pcbi.1006101.s004.tif]

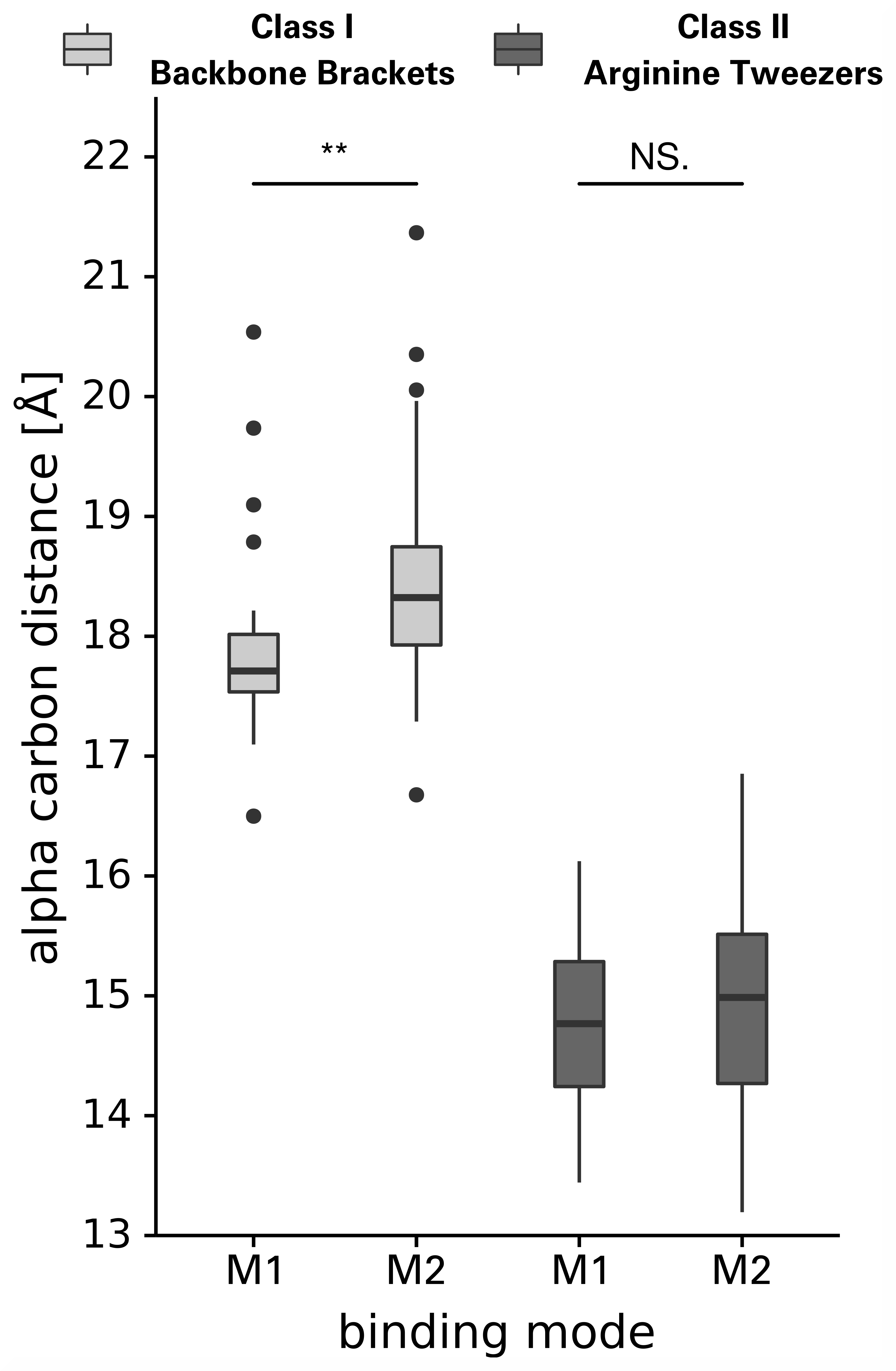

Supplement: S5 Fig — Distributions of alpha carbon distances for Class I Backbone Brackets motif and Class II Arginine Tweezers motif in adenosine phosphate bound (M1) and unbound state (M2). The alpha carbon distance of the Backbone Brackets differs significantly between the two states (Mann-Whitney U p<0.01). (TIF) [file pcbi.1006101.s005.tif]

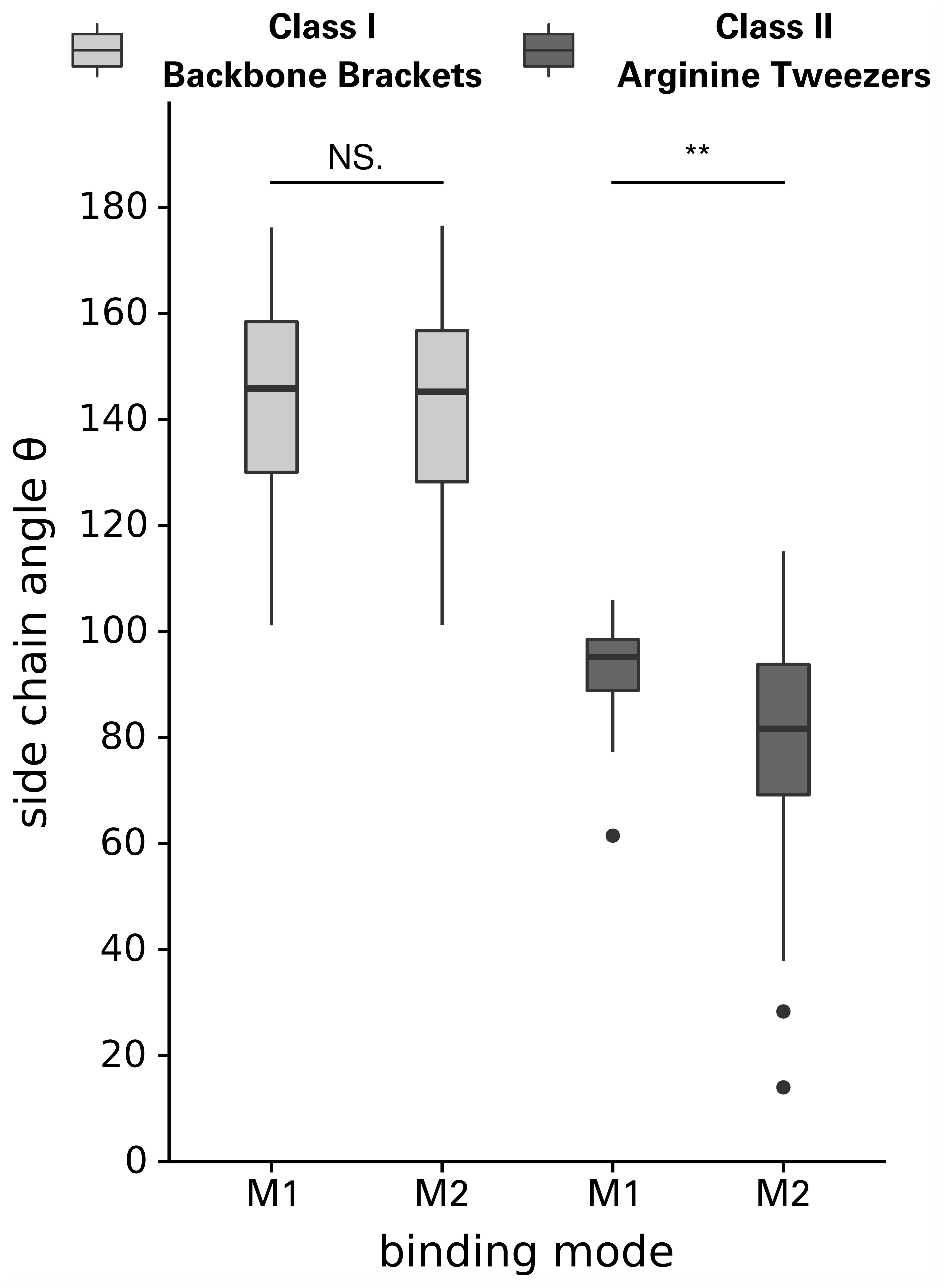

Supplement: S6 Fig — Distributions of side chain angle θ for Class I Backbone Brackets motif and Class II Arginine Tweezers motif in adenosine phosphate bound (M1) and unbound state (M2). The side chain angles of the Arginine Tweezers differs differs significantly between the two states (Mann-Whitney U p<0.01). (TIF) [file pcbi.1006101.s006.tif]

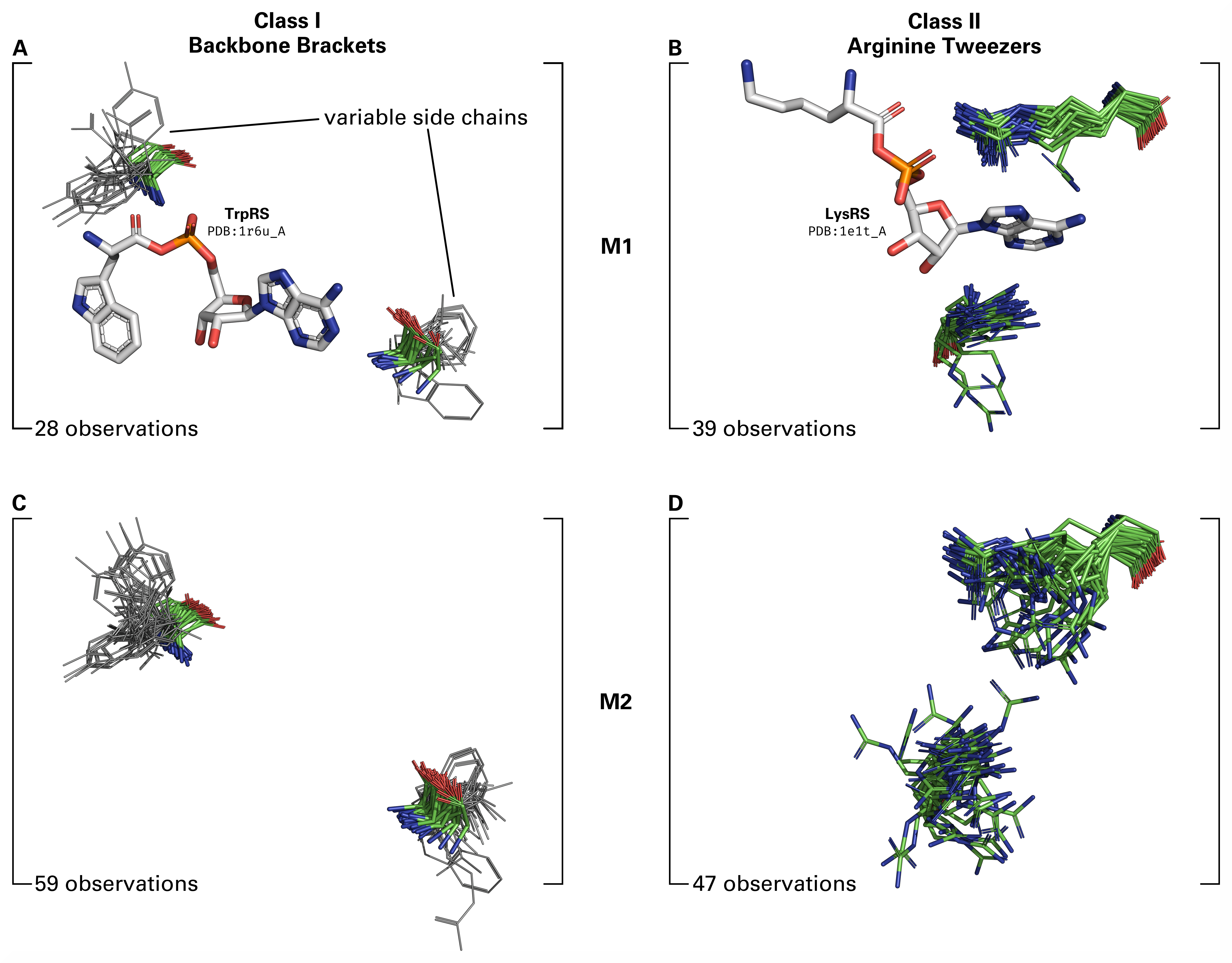

Supplement: S7 Fig — Structural backbone-only alignments of relevant binding site motifs computed with Fit3D [122]. Alignments are grouped by structures derived from adenosine phosphate bound (M1) and unbound state (M2) for aaRS Class I and Class II. (A,C) The Class I Backbone Brackets motif aligned in respect to M1 and M2. A high side chain variance (gray line representation) is evident if an adenosine phosphate ligand is bound (A) and if the ligand is absent (C). However, backbone orientations are highly conserved to realize consistent hydrogen bond interaction with the adenosine phosphate part of the ligand. (B,D) The Class II Arginine Tweezers motif aligned in respect M1 and M2. Low side chain variance can be observed if an adenosine phosphate ligand is bound (B), whereas the absence of an adenosine phosphate ligand (D) allows an increased degree of freedom for side chain movement. Averaged backbone and side chain RMSD values after all-vs-all superimposition are shown in S1 Table. (TIF) [file pcbi.1006101.s007.tif]

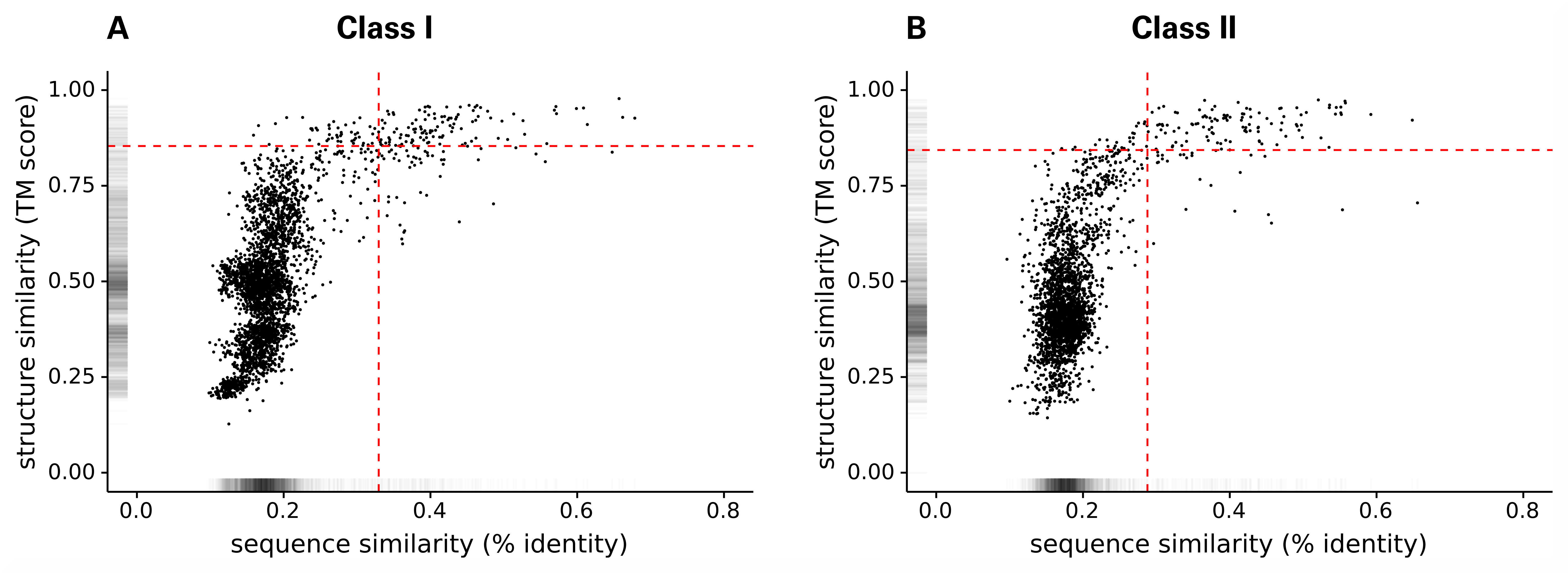

Supplement: S8 Fig — Structure and sequence similarity for pairs of cluster representative chains for aaRS Class I (A) and II (B). Depicted is the sequence similarity (% identity) after a global Needleman-Wunsch [121] alignment of both structures against the structure similarity determined by TMAlign [73]. For Class I (Class II) 95% of all pairs exhibit <33% (29%) sequence identity and <0.85 (0.84) TM score. The 95% quantile borders are depicted as red dashed lines. (TIF) [file pcbi.1006101.s008.tif]

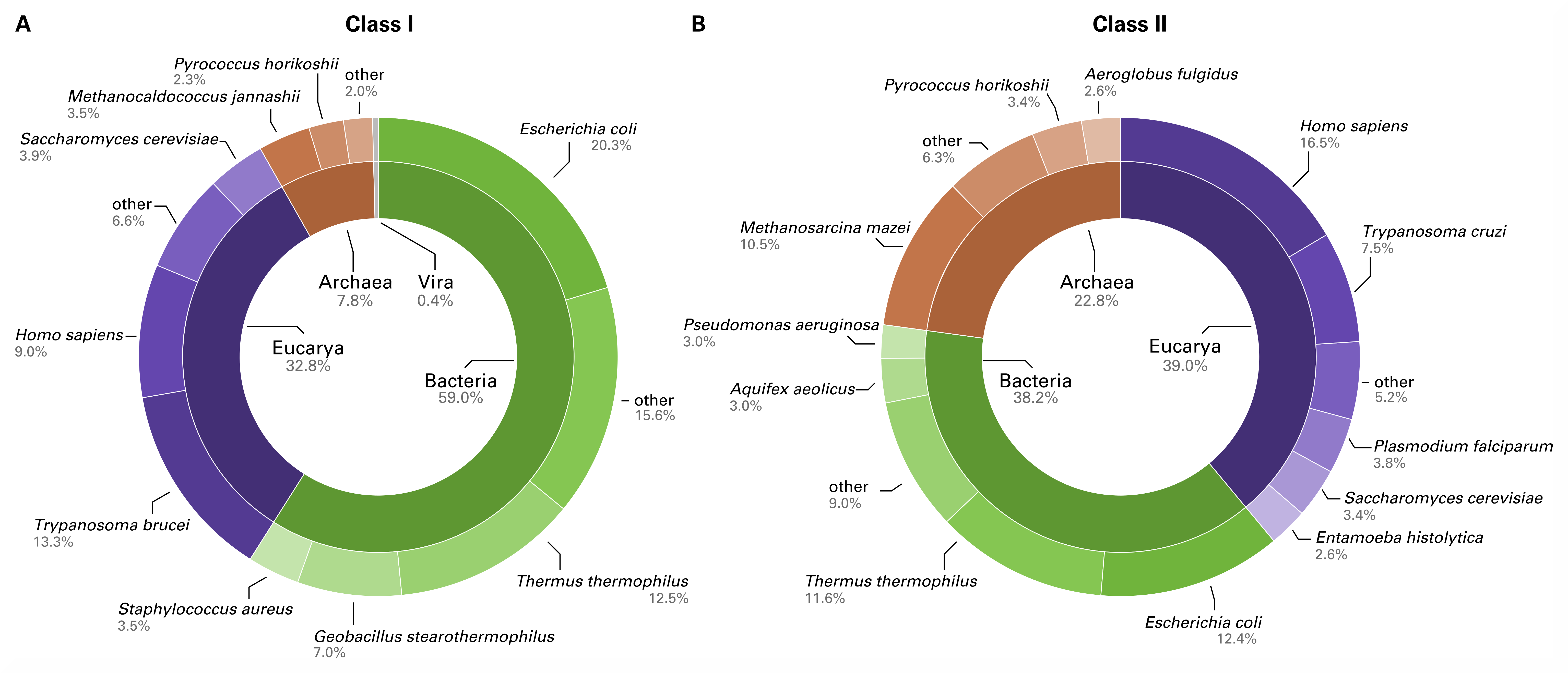

Supplement: S9 Fig — The organisms of origin for aaRS Class I (A) and Class II (B) structures in the dataset. The inner circles correspond to the superkingdom of the organism. The outer circle depicts the partition into specific species (combining different strains). Sections representing eukaryotic species are colored in violet, bacteria are colored in green, archaea are colored in orange and vira are colored in gray. Species, that are origin of less than two percent of the structures are condensed to the “other” cluster for each superkingdom. All superkingdoms are represented in both datasets. Class I contains more bacterial structures than Class II, but fewer originating from eukaryotes or archaea. Interestingly, Class I also contains one viral structure. The Class I set contains four mitochondrial structures, whereas Class II contains 15 mitochondrial structures. Despite the diverse origins of the structures the conserved interaction patterns can be observed. (TIF) [file pcbi.1006101.s009.tif]
