# Supplementary material for: Backbone Brackets and Arginine Tweezers delineate Class I and Class II aminoacyl tRNA synthetases
Source: PLoS Comput Biol. 2018 Apr 16;14(4):e1006101. doi: 10.1371/journal.pcbi.1006101 (PMC5919687; doi:10.1371/journal.pcbi.1006101)
Supplement: S1 Table — Averaged backbone RMSD values after all-vs-all superimposition are shown in this table. (DOCX) [file pcbi.1006101.s010.docx]

## S1 Table: Backbone RMSD of Backbone Brackets and Arginine Tweezers after superimposition

| **motif** | **binding mode*** | **observations** | **backbone RMSD [Å]** |  |
| --- | --- | --- | --- | --- |
| Backbone Brackets | M1 | 28 | 0.32 |  |
| Backbone Brackets | M2 | 59 | 0.34 |  |
| Arginine Tweezers | M1 | 39 | 0.24 |  |
| Arginine Tweezers | M2 | 47 | 0.28 |  |
|  |  |  |  |  |
| *M1 contains an adenosine phosphate ligand, no adenosine phosphate ligand in M2. | | | | |
