# Supplementary material for: Backbone Brackets and Arginine Tweezers delineate Class I and Class II aminoacyl tRNA synthetases
Source: PLoS Comput Biol. 2018 Apr 16;14(4):e1006101. doi: 10.1371/journal.pcbi.1006101 (PMC5919687; doi:10.1371/journal.pcbi.1006101)
Supplement: S1 Appendix — (DOCX) [file pcbi.1006101.s011.docx]

## S1 Appendix: Dataset preparation

All selected protein chains from the PDB carry one of the following protein family annotations, according to Pfam [1]: PF00133, PF00152, PF00579, PF00587, PF00749, PF00750, PF01406, PF01409, PF01411, PF02091, PF02403, PF02912, PF03485, PF09334. Additionally, structures annotated with an EC number indicating tRNA-ligation activity were selected: 6.1.1.1 (TyrRS), 6.1.1.2 (TrpRS), 6.1.1.3 (ThrRS), 6.1.1.4 (LeuRS), 6.1.1.5 (IleRS), 6.1.1.6 (LysRS), 6.1.1.7 (AlaRS), 6.1.1.9 (ValRS), 6.1.1.10 (MetRS), 6.1.1.11 (SerRS), 6.1.1.14 (GlyRS), 6.1.1.15 (ProRS), 6.1.1.16 (CysRS), 6.1.1.17 (GluRS), 6.1.1.18 (GlnRS), 6.1.1.19 (ArgRS), 6.1.1.20 (PheRS), 6.1.1.21 (HisRS), 6.1.1.22 (AsnRS), 6.1.1.23 (AspRS), 6.1.1.26 (PylRS).

For each of the resulting structures, the existence of a catalytic domain was checked manually and only the chains containing a domain with confirmed catalytic activity were retained. If there were ligands present in the structure, these ligands were annotated manually to avoid errors in the assignment of ligands to their catalytic chain. This procedure resulted in a high-quality dataset of 972 individual aaRS chains containing a catalytic domain.

[1] Finn RD, Coggill P, Eberhardt RY, Eddy SR, Mistry J, Mitchell AL, *et al.*

The Pfam protein families database: towards a more sustainable future.

*Nucleic Acids Res.* 2016;44(D1):D279--285.
