# Supplementary material for: Backbone Brackets and Arginine Tweezers delineate Class I and Class II aminoacyl tRNA synthetases
Source: PLoS Comput Biol. 2018 Apr 16;14(4):e1006101. doi: 10.1371/journal.pcbi.1006101 (PMC5919687; doi:10.1371/journal.pcbi.1006101)
Supplement: S2 Appendix — (DOCX) [file pcbi.1006101.s012.docx]

## S2 Appendix: Selection of representative entries

In order to avoid redundancy, representatives were defined for each sequence cluster with >95% sequence identity and discriminated between three types: cluster representatives, representatives that contain an adenosine phosphate ligand (M1), and representatives that do not contain an adenosine phosphate ligand (M2).

The selection criteria for these categories were defined as follows:

- cluster representative:
  1. protein must be wild type (if wild type exists)
  2. best resolution
  3. longest sequence coverage
- representatives with an adenosine-relevant ligand
  1. chain must contain an adenosine phosphate ligand
  2. this ligand must be standard (adenosine phosphate or close derivate)
  3. no experimentally validated inhibitor ligand in the binding site
  4. protein must be wild type (if wild type exists)
  5. best resolution
- representatives without an adenosine phosphate ligand
  1. chain must not contain an adenosine phosphate ligand
  2. binding site must not contain an inhibitor ligand
  3. protein must be wild type (if wild type exists)
  4. best resolution

For ties in the selection, structures were sorted naturally ascending according to their PDB identifier and chain identifier and the first was chosen.
